# High-Moisture Extrusion of Mixed Proteins from Soy and Surimi: Effect of Protein Gelling Properties on the Product Quality

**DOI:** 10.3390/foods11101397

**Published:** 2022-05-12

**Authors:** Yujie Zhang, Jinchuang Zhang, Qiongling Chen, Ning He, Qiang Wang

**Affiliations:** 1 Institute of Food Science and Technology, Chinese Academy of Agriculture Sciences, Key Laboratory of Agro-Products Processing, Ministry of Agriculture and Rural Affairs, Beijing 100193, China; zhangyujie1973@163.com (Y.Z.); zhangjinchuang1002@163.com (J.Z.); cql_ttxs@163.com (Q.C.); 2 Department of Chemical and Biochemical Engineering, College of Chemistry and Chemical Engineering, Xiamen University, Xiamen 361001, China; chem-xmu@xmu.edu.cn

**Keywords:** soy protein isolate, surimi, gelling properties, high-moisture extrusion, texture

## Abstract

The high-moisture extrusion of proteins from plant and animal sources should be a new way for developing alternative protein products with meat-like texture. The protein gelling properties are considered an important factor for the meat-like texture formation during the high-moisture extrusion processing. In this study, the mixed protein gelling properties from soy protein isolate (SPI) and surimi at different ratios (90:10, 80:20, 70:30, 60:40 and 50:50) were investigated to relate to the high-moisture (70%) extruding product textural properties, correspondingly. Results showed that at SPI–surimi ratio 60:40, the heat-induced gelation time was clearly extended and the gel strength became much weaker. During the high-moisture extrusion processing, at SPI–surimi ratio 80:20, the extrudate showed the higher hardness, chewiness, gel strength and fibrous degree, while excessive surimi (more than 40%) in the blends would hinder the fibrous-oriented structure formation. It suggested that SPI may act as the continuous phase that is dispersed by surimi during the high-moisture extrusion processing. Interestingly, it was found that the gel strength of SPI–surimi blends was nonlinearly correlated with the specific mechanical energy (SME) and product textural properties. The study would be helpful for improving the textural properties of alternative protein products from soy and surimi.

## 1. Introduction

The global population is expected to reach about 9.5 billion by 2050, resulting in the rapid rise of global demand for proteins [1,2]. Likewise, as predicted by the Food and Agriculture Organization (FAO) of the United Nations, global demand for animal meat would reach 455 M metric tons by 2050 (a 76% increase from 2005) [3]. Nevertheless, the production of animal meat, especially livestock meat, is unsustainable, consuming fresh water and farmland, discharging masses of greenhouse gas and causing severe environmental impacts [4,5,6]. In this case, multitudinous meat substitute products produced by sustainable protein resources, such as soy protein, have been exploited, and the marketplace is expected to grow at a rate of 7.9% from 2019 to 2024 [7]. Nowadays, to gain the sufficient nutrition (e.g., complementary amino acids profiles) and desirable sensory properties of meat substitute products, mixed plant and animal proteins have attracted growing interest [8]. Surimi, one of the important products of fish resource processing and utilization, having high protein content, low lipid content and being rich in polyunsaturated fatty acids, is a promising animal protein source [9]. Further, some new meat substitute products with novel textural and sensory properties can be exploited by mixing the surimi protein with plant proteins [10].

Extrusion technology has been used to process the blends of soy and surimi protein to obtain the meat-like texture [11,12]. He et al. [13] extruded the hair tail surimi and SPI blends and found that the barrel temperature significantly affected the water-holding capacity of the extrudates, while the texture properties of the extrudates were determined by the feed moisture. Moreover, the in vitro protein digestibility of the blends was improved by the extruding process. Recently, high-moisture extrusion technology has attracted growing attention, due to its high efficiency, lower energy input and no waste discharge [14,15]. The protein molecules underwent unfolding, associating, aggregating, and cross linking during high-moisture extrusion processing, to finally form the fibrous-oriented structure [16]. Thiébaud et al. [12] obtained the layered thin fibrous structure of surimi and soy protein concentrate blends through high-moisture extrusion technology. It was also found that the textural properties of the extrudates were closely related to the barrel temperature, feed moisture, and the screw speed. However, during the high-moisture extrusion processing, little is known about the effect of protein gelling properties from soy and surimi on the textural properties of the final extrudates.

During thermomechanical processing, it is well known that protein gelling properties play an important role in the final quality formation [17]. Luo et al. [18] reported that mixing soy protein isolate (SPI) with Alaska pollock surimi would lead to a weaker gel matrix, resulting in a decreased breaking force and a lower lightness in the final gel sausage. Park [19] found that a 1% content of SPI added to the surimi would help to form a more rigid structure, which increased the shear stress and decreased the shear strain when gelling. Recently, researchers have focused on enhancing the gelling properties by modifying the protein molecules to further improve the qualities [20,21]. Ramírez-Suárez et al. [22] indicated that after being modified by the microbial transglutaminase, the myofibrillar protein showed a better gelling ability when mixing with SPI. Guo et al. [23] found that when mixing the myofibrillar protein with soy protein, a denser and more orderly protein network was formed, resulting in a significant improvement in water-holding capacity. Thus, to develop alternative protein products with desirable texture from plant and animal proteins, the protein gelling properties should play an essential role.

Therefore, the purposes of this research are to process SPI–surimi blends at different ratios using the high-moisture extrusion method; to characterize the gelling properties of mixed proteins using rheometer and texture analyzers; to evaluate the textural properties and macro- and micro-structure of the high-moisture extrudates; and to build the relationships among the gelling properties of the mixed proteins, the fibrous degree, hardness, chewiness, and gel strength of the extrudates, and the specific mechanical energy (SME) by polynomial regression curve fitting.

## 2. Materials and Methods

### 2.1. Materials

SPI was supplied by Yihai Kerry Co., Ltd. (Shanghai, China). Surimi (Nemipterus virgatus) was supplied by Fujian Anjoy Foods Co., Ltd. (Xiamen, China) and could be stored for 1 year. As shown in Table S1, the protein content (dry basis) of SPI and surimi were 90.81% and 52.78%, respectively. According to the preliminary experiment and considering the extrudability of the extruder, the SPI and surimi were mixed at different ratios (90:10, 80:20, 70:30, 60:40, 50:50) using a mixer (JHF-20L, Zhengzhou Jinhe Machinery Manufacture Co., Ltd., Zhengzhou, China). 

### 2.2. Rheological Measurements

Protein blends in Section 2.1 were reconstituted at 10% (*w*/*w*) in deionized water stirring for 2 h, and then stored overnight at 4 °C. The rheological properties of the blends were tested by a Discovery HR-2 rheometer (TA Instruments Ltd., New Castle, Germany) equipped with parallel plates (diameter of 40 mm; gap of 1 mm) and the equilibrium time was 5 min. During the steady shear tests, the viscosity was determined at 25 °C and the shear rate rage was 1–100 s^−1^ [24]. In the dynamic oscillatory tests, the strain was 0.5% and the samples were kept for 2 min and heated at a rate of 2 °C/min from 25 °C to 90 °C, and then kept for 30 min at 90 °C before cooling to 25 °C at 2 °C/min [25]. A thin layer of silicone oil was used for the edges of the samples to prevent evaporation. The storage modulus (G′) and loss modulus (G′′) were recorded and the tanδ was calculated by the ratio the G′′ and G′.

### 2.3. Protein Gelling Properties

Five groups of the SPI–surimi blends mentioned in Section 2.1 were mixed with deionized water at a concentration of 30% and stuffed into the casing (diameter 22 mm), then kept in 40 °C water bath for 30 min. After this, the samples were heated to 90 °C for 30 min and placed into ice water immediately to prepare the gel. To equilibrate sufficiently, the conditioned gel was kept at 4 °C overnight. According to the method of Niu et al. [26], the gel strength was measured by a TA.XT2 Texture Analyzer (Stable Micro Systems, Godalming, UK) with a P/0.5 probe (diameter 12.7 mm). The heat-induced or extruded protein gel samples were cut into tiny cylinders with a length of 15 mm. The compress speed was 1 mm·s^−1^ and the gel strength was reflected by the forces when the sample was punctured to a depth of 4 mm; at least ten parallel samples were measured for each sample.

### 2.4. High-Moisture Extrusion Experiments

The extruding experiments were carried out by a twin-screw extruder (FMHE36-24, FUMACH, China) with a screw diameter of 36 mm and a length/diameter ratio 24:1. At the exit of the extruder barrel, a long cylindrical cooling die with a diameter of 22 mm was attached. The extruder barrel was segmented into a feeding zone and five temperature-controlled zones, which were heated by an electric cartridge heating system and cooled with running water. During high-moisture extrusion processing, the feed speed of the protein blends in Section 2.1 was set at 7 kg/h. The feed moisture was adjusted to 70% (*w*/*w*) online (the same with the water content for preparing the heat-induced gels), the screw speed was 210 rpm, and the extruder barrel temperatures were kept at 60, 90, 135, 135, and 110 °C from the first zone to the fifth zone, respectively. The cooling die was kept at 50 °C controlled by the running water. At least ten parallel samples were made for each sample of extrudates.

### 2.5. Specific Mechanical Energy (SME)

The specific mechanical energy (*SME*, kJ·kg^−1^) was calculated from the screw speed (*n*, rpm), motor torque (*T*, N·m) and mass flow rate (*MFR*, g·min^−1^), according to the following formula [27], and at least ten parallel samples were measured for each sample:

(1) $$SME=\frac{2\pi \times n\times T}{MFR}$$

### 2.6. Textural Properties of the Extrudates

The textural properties of the extrudates were measured by the TA.XT2 Texture Analyzer (Stable Micro Systems, UK). The extrudates at a length of 10 mm were compressed with a P/36R probe (cylinder, *Φ* 36 mm) to 50% of their original thickness at a speed of 1 mm·s^−1^ and the hardness and chewiness of the extrudates were recorded. The crosswise shear force (FV, the sample was cut along the vertical direction) and the lengthwise shear force (FL, the sample was cut parallel to the extruding direction) were determined with an A/CKB probe (knife blade). The pre-test speed during experiment was chosen as 2 mm·s^−1^, the test speed was kept at 1 mm·s^−1^, the post-test speed was 2 mm·s^−1^, and the strain was 75%. The fibrous degree was expressed by the ratio FV to FL [28]. At least ten parallel samples were measured for each sample.

### 2.7. Macro- and Microstructure Detection

The macrostructure images of the extrudates were taken by the camera (D90, Nikon, Tokyo, Japan). The microstructure of the extrudates was examined by a scanning electron microscopy (SU8010, Hitachi, Japan). The fresh extrudates were cut into tiny cuboids with a length of 5 mm and mounted in a glutaraldehyde solution (2.5 vol %) for 48 h. The immobilized extrudates were critical point dried in CO_2_. Dehydrated samples were coated with gold particles with a sputter coater (IB-5, Hitachi Ltd., Tokyo, Japan). The images were taken with the scanning electron microscope at ×3000 [27].

### 2.8. Statistical Analysis

All data were analyzed using analysis of variance (ANOVA) using the Statistical Product and Service Solutions software (version 19.0, SPSS Inc., Chicago, IL, USA). The comparisons between treatments were evaluated using Duncan’s test. Statistical significance was set at a 0.05 probability level. Polynomial regression curve fitting method was used to relate the gelling properties of the blends, SME and the qualities of the extrudates.

## 3. Results and Discussion

### 3.1. Rheological Properties of the SPI–surimi Blends

**Figure 1:**
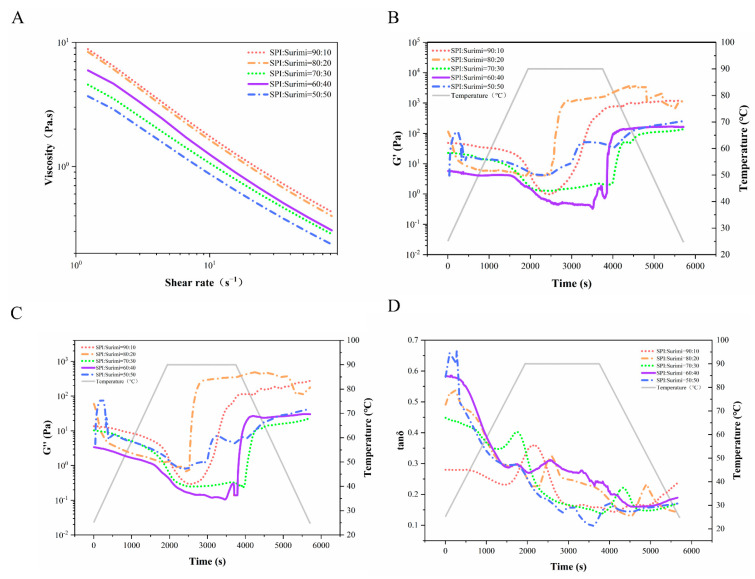
The viscosity (**A**), the thermal profiles in terms of storage modulus (G′) (**B**), loss modulus (G″) (**C**) and tanδ (**D**) in temperature ramp for the SPI and surimi blends at different ratios.

As shown in Figure 1A, when increasing the shear rate, the apparent viscosity of all samples clearly decreased, attributed to the destruction of the intra- or intermolecular protein–protein interactions [29]. At SPI–surimi ratio 50:50, the apparent viscosity was obviously lower, while it clearly increased when the SPI content was higher than 80%. This could be explained by the fact that the S-S bonds would form during soy protein purification, resulting in the formation of protein aggregates at SPI content ≥ 80% and, thus, lead to larger shear resistance [8]. Moreover, myosin (the major components of surimi protein) showed a characteristic rod-like chain, which is more orientated and vulnerable when shearing, contributing to a lower apparent viscosity at surimi content ≥ 30% [30,31].

Figure 1B,C showed the changes in G′ and G′′ of SPI–surimi blends, respectively, on temperature ramp. When increasing the temperature, the G′ and G′′ decreased firstly due to the unfolding of protein molecular chains, especially at SPI–surimi ratio 80:20 [26]. This indicated the enhancement of the protein molecular chains disentanglement with more SPI under heating and shearing [32]. Interestingly, at SPI–surimi ratio 50:50, an obvious peak appeared when increasing the temperature from 30 °C to 50 °C, which should be related to the surimi protein–protein molecular interactions, such as the degradation of myosin, the dissociation of actin–myosin, and the denaturation of myosin tail [33]. The G′ and G′′ increased obviously as the temperature gradually reached 90 °C, especially for the samples with a higher SPI content (≥80%), which could be due to the protein molecular aggregating and cross linking [34,35]. In addition, the gel started to form at a similar time (the time at the lowest G′) at SPI–surimi ratios 90:10, 80:20, and 70:30, respectively, while the gelation time was markedly prolonged at SPI–surimi ratio 60:40. This suggested that the gelling phenomenon was clearly delayed at a higher surimi ratio, contributing to form a weaker gel [36]. At SPI–surimi ratio 90:10 and 80:20, the initial, maximal, and the final G′ and G′′ of the blends were obviously higher, which was consistent with Wang et al. [21], who also found that the addition of heat-treated SPI greatly improved the final G′ of the mixed gels with the myofibrillar protein. This suggested that under heating and shearing, at SPI content ≥ 80% in the blends, the unfolding of the protein molecular chains and the protein–protein molecular interactions were both enhanced, leading to a higher flexibility in the protein molecular chains and, thus, forming a stronger gel [22]. On the contrary, at surimi content ≥ 30% in the blends, the increase in the G′ and G′′ was hindered when heating and shearing, resulting in a lower flexibility of the protein molecular chains, which tended to form a weaker gel. 

Figure 1D shows that the tanδ values of all samples were lower than one and clearly decreased under heating and shearing, suggesting solid-like behavior of the blends and the transformation from a weak gel to a more rigid gel structure [37,38]. This could be due to that the heating and shearing breaking some physical interactions. At the same time, some interactions with a lesser quantity but more stable energy could be formed. When the temperature increased to 80 °C, obvious peaks appeared due to the structural transitions of the protein molecules. Interestingly, the peak became sharper as the SPI content ≥ 70% in the blends, confirming that higher SPI content was beneficial to the protein molecular unfolding and cross linking, which lead to an interdigitated network and firm gel [39]. 

### 3.2. Heat-Induced Gelling Properties of the SPI–surimi Blends

**Figure 2:**
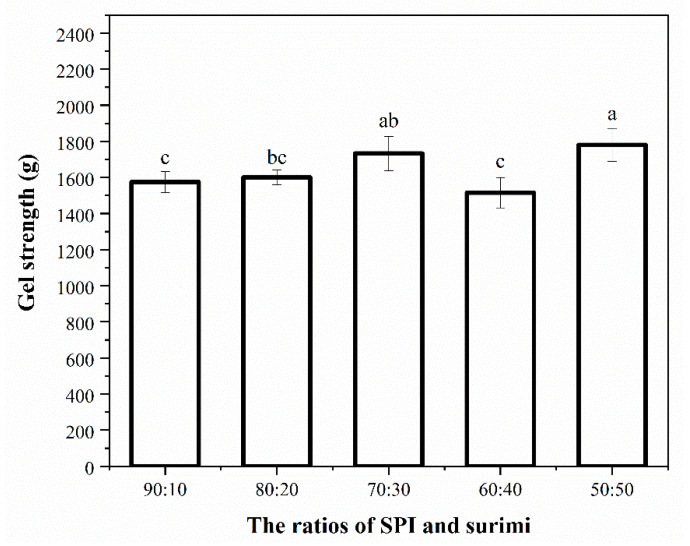
The heat-induced gel strength of the SPI and surimi blends at different ratios. Different letters mean significant differences (*p* < 0.05).

In Figure 2, at SPI–surimi ratio 50:50, the heat-induced gel showed the highest gel strength. This indicated that decreasing the SPI content in the blends was helpful for improving the heat-induced gel strength [18,26]. These interesting data were consistent with the obvious peak that appeared on the G′ and G′′ curves at SPI–surimi ratio 50:50 from 30 °C to 50 °C due to the surimi protein–protein molecular interactions, which increased the gel strength. Additionally, the unfolding and cross linking of the SPI protein molecules may be insufficient at the set heating cycle and then prevent the aggregating of the surimi myosin heavy chains when heating at 40 °C, consequently resulting in a weak gel when increasing the SPI content [18,32,40]. Furthermore, the lower gel strength at SPI–surimi ratio 60:40 may also be due to the extension of the gelling time, according to the rheological results.

### 3.3. Textural Properties of the SPI–surimi High-Moisture Extrudates

As shown in Table 1, at SPI–surimi ratio 90:10 and 80:20, the hardness, chewiness and gel strength of the extrudates were improved significantly (*p* < 0.05) but decreased significantly (*p* < 0.05) when the surimi content increased to 50%, which was completely different from the heat-induced gel. This was mainly due to the fact that during the high-moisture extrusion processing, the total protein concentration in the blends may play an important role for the textural properties of the extrudates [41]. Moreover, the blends with more surimi possessed better water-holding capacity [42], which may prevent the protein aggregating during the high-moisture extrusion processing, thus, resulting in lower hardness, chewiness and the gel strength of the extrudates, which may contribute to a softer and shriveled final product. The crosswise strength, lengthwise strength and the fibrous degree of the extrudates all decreased significantly (*p* < 0.05) when increasing the surimi content higher than 40%, showing that the arrangement of the fibers parallel to the extrusion direction could be hindered with a higher surimi content in the blends, leading to a lower fibrous degree, which may lead to a looser final product [43].

### 3.4. Macro- and Microstructure of the SPI–surimi High-Moisture Extrudates

**Figure 3:**
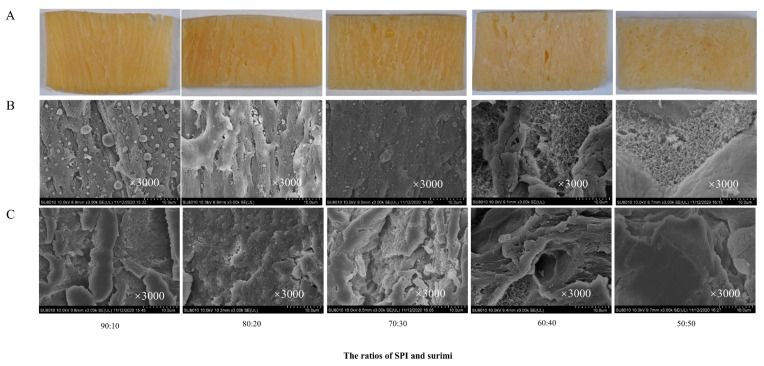
The macro- (**A**) and microstructure (**B**,**C**) of the extrudates of the SPI and surimi blends at different ratios along the extrusion direction (**A**,**B**) and perpendicular to the extrusion direction (**C**)**.**

Figure 3A,B display the macrographs and SEM micrographs taken from the sections along with the extrusion direction. It could be seen that the phase separation phenomenon and oriented fibrous structure were found in the extrudates at surimi content less than 30%, in which the surimi particles filled into the SPI gel. This phenomenon became more obvious when further decreasing the surimi content in the blends. Interestingly, with a higher surimi content (40% or 50%), the surimi particles disappeared gradually and a loose protein network with non-orientation was formed, embedded in the SPI gel, leading to the formation of a spongy structure. The results showed that when increasing the surimi content higher than 40%, the interactions between soy protein and surimi protein molecules became weaker, while the protein–protein interactions of surimi were enhanced to form the partial network [44]. In Figure 3C, on the surface perpendicular to the extrusion direction, the obvious s layers with a certain orientation were found at surimi content less than 30%. When increasing the surimi content to higher than 40%, the structure became looser and disordered. 

The microstructure results suggested that during the high-moisture extrusion processing, the SPI should be the continuous phase and surimi may act as the dispersed phase [26]. The surimi particles filled into the SPI gel and led to a rigid structure with a higher fibrous degree [45]. However, excessive surimi would obviously hinder the formation of the oriented layer structure, which further affected the formation of the fibrous structure, resulting in the formation of weak and viscous extrudates with a higher SME (seen in Section 3.5). This was also consistent with the results for the textural properties.

### 3.5. Specific Mechanical Energy (SME) during the High-Moisture Extrusion Process

**Figure 4:**
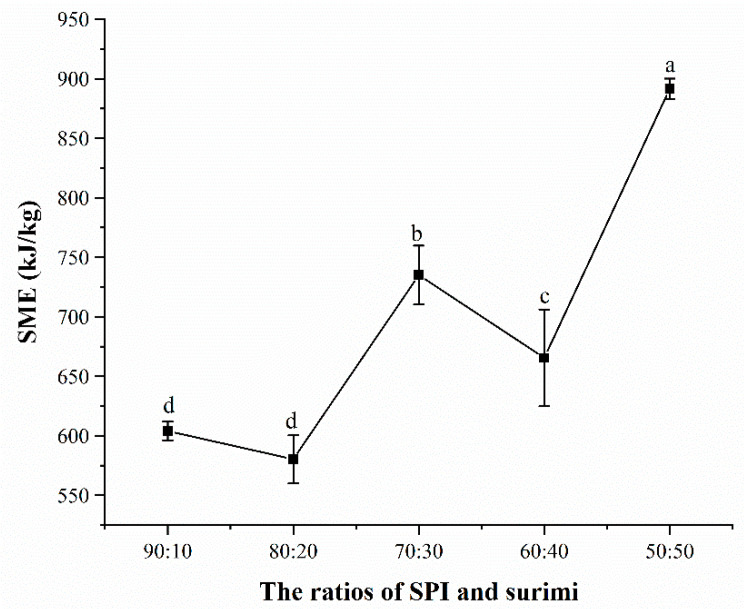
SME during the high-moisture extrusion processing of the SPI and surimi blends. Different letters mean significant differences (*p* < 0.05).

The SME increased significantly (*p* < 0.05) when increasing the surimi content in the blends during the high-moisture extrusion processing, as shown in Figure 4. It seems that the blends with a higher surimi ratio tended to form a gel with lower strength and looser structure that decreased the degree of the barrel fill, thus, leading to a lower mass flow rate of the melt and a higher SME [46,47]. Moreover, the blends containing a higher surimi ratio demonstrated a higher water-holding capacity, which also contributed to increasing the viscosity and energy dissipation of the blends during the high-moisture extrusion processing [27,42]. 

### 3.6. Effect of the Heat-Induced Gel Strength of the Blends on the SME

**Figure 5:**
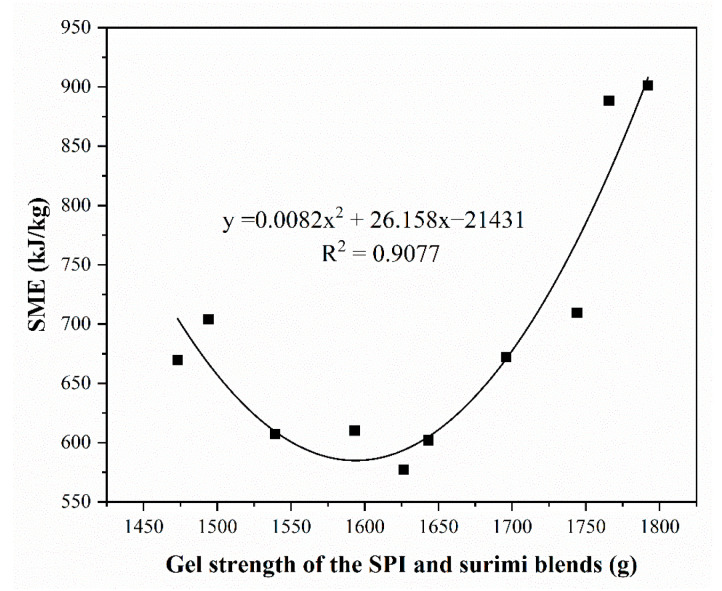
Relationship between the gel strength of the SPI and surimi blends and the SME.

As shown in Figure 5, the polynomial regression fitting result determined the relationship between the gel strength of the SPI–surimi blends and the SME during high-moisture extrusion processing. The R^2^ was 0.9077, which indicated that the gel strength of the SPI–surimi blends correlated very well with the SME. In the range investigated, the increase in the gel strength firstly reduced the SME and then obviously enhanced the SME. This result suggested that a proper gel strength (lower than 1600 g) of the SPI–surimi blends should be beneficial to the energy conservation during the high-moisture extrusion processing.

### 3.7. Effect of the Heat-Induced Gel Strength of the Blends on Textural Properties of the Extrudates

**Figure 6:**
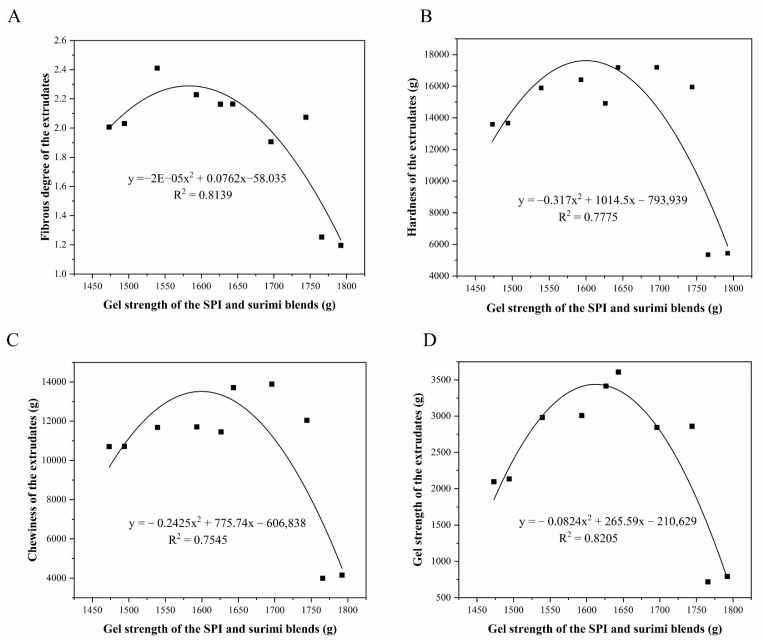
Relationships between the gel strength of the SPI and surimi blends and the fibrous degree (**A**), hardness (**B**), chewiness (**C**) and gel strength (**D**) of the extrudates.

Figure 6 displays the polynomial regression fitting results for the gel strength of SPI–surimi blends with the fibrous degree, hardness, chewiness, and gel strength of the extrudates. It was found that the gel strength of the SPI–surimi blends was not linearly related with the fibrous degree, hardness, chewiness, and gel strength of the extrudates. The R^2^ of the fitting curves were 0.8139, 0.7775, 0.7545, and 0.8205, correspondingly, which suggested that the gel strength of the SPI–surimi blends correlated very well with the fibrous degree, hardness, chewiness, and the gel strength of the extrudates. In the investigated range, with the enhancement of the gel strength, the fibrous degree, hardness, chewiness, and the gel strength of the extrudates increased firstly and then decreased obviously. Thus, to improve the textural properties of extrudates containing soy and surimi proteins, the gel strength should be controlled within a reasonable range, which should not be higher than 1600g. This was attributed to the fact that a stronger gel strength in the soy and surimi protein blends would hinder the protein unfolding during the high-moisture extrusion processing, thus, leading to the poor textural properties of the extrudates. 

### 3.8. Effect of the SME on Textural Properties of the Extrudates

**Figure 7:**
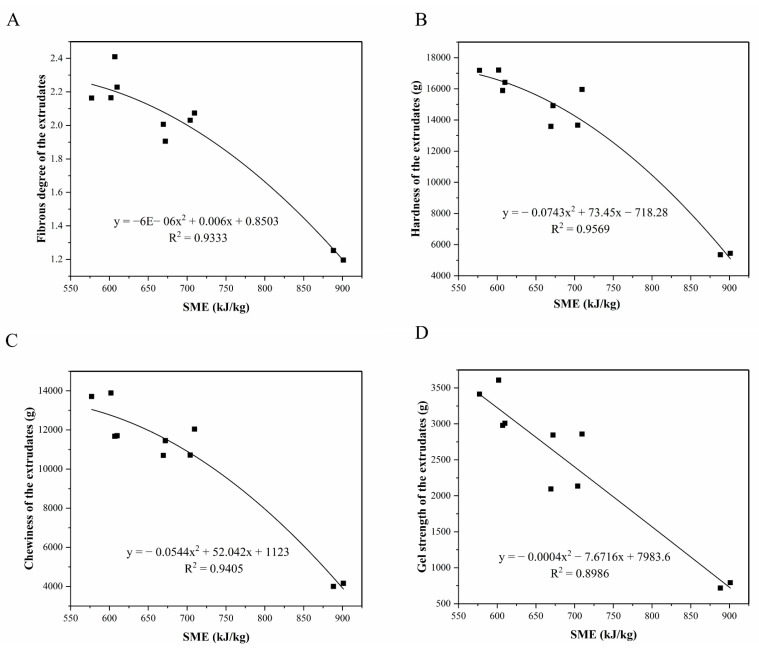
Relationships between the SME and the fibrous degree (**A**), hardness (**B**), chewiness (**C**) and gel strength (**D**) of the extrudates.

Figure 7 shows the polynomial regression fitting results of the SME with the fibrous degree, hardness, chewiness, and the gel strength of the extrudates. The R^2^ of the fitting curves were 0.9333, 0.9569, 0.9405, and 0.8986, respectively, indicating that the SME correlated very well with the fibrous degree, hardness, chewiness, and gel strength of the extrudates. In the investigated range, increasing the SME, the fibrous degree, hardness, chewiness, and gel strength of the extrudates decreased obviously, which indicated that the SME had a negative impact on the textural properties of the extrudates. These results were consistent with the conclusion of Zhang et al. [43], who also found that the SME was negatively related with the fibrous degree of soy protein and starch-mixed extrudates.

## 4. Conclusions

When heating and shearing, the storage modulus (G′) of the SPI and surimi protein blends decreased obviously as the surimi ratio increased to 50%. At SPI–surimi ratio 60:40, the gelation time was clearly extended and the heat-induced gel strength became much lower. However, the heat-induced gel strength was enhanced at SPI–surimi ratio 50:50 due to the surimi protein–protein molecular interactions. During the high-moisture extrusion processing, at SPI–surimi ratio 80:20, the extrudate showed the highest hardness, chewiness, gel strength and fibrous degree, which may lead to the juicy and tender product taste of meat. However, excessive surimi (more than 40%) in the blends hindered the formation of the fibrous-oriented structure in the extrudates. It suggested that SPI should be the continuous phase and the surimi may act as the dispersed phase when forming the fibrous-oriented structure during the high-moisture extrusion processing. Interestingly, the heat-induced gel strengths of the SPI–surimi blends were nonlinearly correlated with the SME and textural properties of the extrudates. It was also found that the SME was negatively related to the fibrous degree, hardness, chewiness, and gel strength of the extrudates. The study has provided the information that textural properties of the extrudate containing soy and surimi proteins can be improved by changing the gelling properties of the raw material, which was also helpful for developing alternative protein products from soy and surimi. In future studies, the relationship between the functional properties, such as emulsion and foamability of the mixed surimi and soy protein materials, and the quality of the products should be determined. Further, products with surimi and other plant proteins can be developed.

## Figures and Tables

**Table 1:** Textural properties of the extrudates at different SPI and surimi ratios.

SPI/Surimi	Hardness (N)	Chewiness (N)	Gel Strength (N)	Crosswise Strength (N)	Lengthwise Strength (N)	Fibrous Degree
90:10	16.11 ± 0.42 b	11.70 ± 0.39 b	3.02 ± 0.11 b	0.93 ± 0.04 a	0.40 ± 0.03 ab	2.33 ± 0.10 a
80:20	17.14 ± 0.26 a	13.71 ± 0.26 a	3.50 ± 0.16 a	0.97 ± 0.05 a	0.45 ± 0.01 a	2.16 ± 0.04 ab
70:30	15.97 ± 0.75 b	12.01 ± 0.40 b	2.89 ± 0.09 b	0.83 ± 0.02 b	0.41 ± 0.04 a	2.03 ± 0.17 b
60:40	13.55 ± 0.19 c	10.71 ± 0.03 c	2.19 ± 0.09 c	0.72 ± 0.01 c	0.35 ± 0.02 b	2.05 ± 0.11 b
50:50	5.36 ± 0.12 d	4.08 ± 0.12 d	0.76 ± 0.05 d	0.28 ± 0.01 d	0.23 ± 0.02 c	1.24 ± 0.07 c

Note: Different letters in the same column mean significant differences (*p* < 0.05).

## Data Availability

Data is contained within the article (or Supplementary Material).

## Supplementary Materials

The following supporting information can be downloaded at: https://www.mdpi.com/article/10.3390/foods11101397/s1, Table S1: Chemical components of the SPI and surimi.
