# Supplementary material for: High-Moisture Extrusion of Mixed Proteins from Soy and Surimi: Effect of Protein Gelling Properties on the Product Quality
Source: Foods. 2022 May 12;11(10):1397. doi: 10.3390/foods11101397 (PMC9141467; doi:10.3390/foods11101397)
Supplement: Supplementary file 1 [file foods-11-01397-s001.zip › foods-1706224-supplementary.pdf]

Table S1. Chemical components of the SPI and surimi.

Chemical components	SPI	Surimi
Protein content (dry basis)	90.81±0.13	52.78±1.13
Water content	5.55±0.40	67.97±0.57
Lipid content (dry basis)	0.36±0.24	8.38±0.15
Fiber content (dry basis)	--	--
Ash content (dry basis)	4.67±0.08	1.73±0.09

Note: "--" means undetected. The water content was analyzed by the method of AOAC (AOAC, 2010). The lipid was evaluated by a soxhlet extractor (VELP SER 148/6, Italy). The fiber content was determined by the method of AOAC (AOAC, 2000). The determination of protein content was according to the method of Kjeldahl (FOSS KJELTEC 2300, Denmark) and the ash content was analyzed by the method of Sluiter (Sluiter, et al., 2005).
